# Observation of the effect of hypothermia therapy combined with optimized nursing on brain protection after cardiopulmonary resuscitation: A retrospective case-control study

**DOI:** 10.1097/MD.0000000000037776

**Published:** 2024-04-19

**Authors:** Yan You, Zheng Gong, Yaxu Zhang, Lirong Qiu, Xiahong Tang

**Affiliations:** a The Second Department of Intensive Care Unit, Fujian Provincial Hospital South Branch, Fuzhou, China; b Department of Emergency Medicine, Fujian Provincial Hospital, Fuzhou, China; c Shengli Clinical Medical College of Fujian Medical University, Fujian Medical University, Fuzhou, China; d Fujian Provincial Key Laboratory of Emergency Medicine, Fuzhou, China; e Department of Critical Care Medicine, The Affiliated People’s Hospital of Fujian University of Traditional Chinese Medicine, Fuzhou, China.

**Keywords:** brain protection, cardiac arrest, hypothermia therapy, optimized nursing, retrospective study

## Abstract

This study aimed to investigate the impact of optimized emergency nursing in conjunction with mild hypothermia nursing on neurological prognosis, hemodynamics, and complications in patients with cardiac arrest. A retrospective analysis was conducted on the medical records of 124 patients who received successful cardiopulmonary resuscitation (CPR) at Fujian Provincial Hospital South Branch. The patients were divided into control and observation groups, each consisting of 62 cases. The brain function of both groups was assessed using the Glasgow Coma Scale and the National Institutes of Health Stroke Scale. Additionally, serum neuron-specific enolase level was measured in both groups. The vital signs and hemodynamics of both groups were analyzed, and the complications and satisfaction experienced by the 2 groups were compared. The experimental group exhibited significantly improved neurological function than the control group (*P* < .05). Furthermore, the heart rate in the experimental group was significantly lower than the control group (*P* < .05). However, no significant differences were observed in blood oxygen saturation, mean arterial pressure, central venous pressure, and systolic blood pressure between the 2 groups (*P* > 0.05). Moreover, the implementation of optimized nursing practices significantly reduced complications and improved the quality of life and satisfaction of post-CPR patients (*P* < .05). The integration of optimized emergency nursing practices in conjunction with CPR improves neurological outcomes in patients with cardiac arrest.

## 1. Introduction

Cardiac arrest (CA) is a common life-threatening emergency in the field of emergency medicine.^[1,2]^ It is associated with poor outcomes despite cardiopulmonary resuscitation (CPR) efforts. CA can be triggered by ventricular fibrillation, myocardial infarction, cardiac tamponade, pulmonary embolism, electrolyte abnormalities, drug overdose, and asphyxia.^[3–6]^ With the widespread dissemination and advancements in CPR techniques and advanced life support technologies, the success rate of CPR in patients with CA has significantly improved. However, the overall prognosis remains poor, with a global average survival rate of <10%.^[7]^ According to domestic statistics, around 5–6% of patients with CA survive after CPR. Research findings indicate that around 30% of patients who achieve a return of spontaneous circulation (ROSC) following a cardiac event suffer from a lasting brain injury. Additionally, nearly 50% of patients are discharged with varying levels of neurological deficits.^[8,9]^ Therefore, brain recovery is a critical focus after CPR, and it is essential to explore effective nursing interventions for post-resuscitation brain injury.

Mild therapeutic hypothermia (MTH) has emerged as a new treatment strategy, with current recommendations suggesting lowering the body temperature to 32 to 34 °C within 12 to 24 hours after CA.^[10]^ The feasibility and safety of MTH have been evaluated in several clinical trials, showing a correlation between MTH and improved neurological survival rates in patients with CA.^[11]^ MTH has gradually become an integral part of post-resuscitation brain recovery for patients with CA, yielding certain positive effects. However, there is a lack of clinical studies that combine MTH with optimized emergency care.

In this study, the implementation value of combining both approaches is explored. Through a retrospective analysis of medical records from 124 patients with ROSC after CA, the impact of optimized emergency care combined with MTH on patients’ neurological outcomes, hemodynamics, and complications is investigated.

## 2. Materials and methods

### 2.1. General information

The medical records of 124 patients who achieved ROSC after CA and CPR were retrospectively analyzed at the Fujian Provincial Hospital South Branch from September 2021 to October 2023. This study has passed the ethics review of the Ethics Committee of Fujian Provincial Hospital with the ethics number K2023-08-009.

The inclusion criteria were as follows:

1. age between 14 and 80 years;
2. meeting the diagnostic criteria for CA set by the American Heart Association^[12]^;
3. successful resuscitation with CPR and eligible for a 90-day follow-up after discharge;
4. no pulmonary infection;
5. complete medical records available;
6. average arterial pressure of at least 60 mm Hg in all successfully resuscitated patients.

The exclusion criteria were as follows:

1. infants and children aged 0 to 14 years;
2. patients who failed CPR;
3. patients with malignant tumors;
4. patients with physical disabilities;
5. patients with a history of neurological disorders or psychiatric illnesses;
6. breastfeeding or pregnant women;
7. patients with missing clinical data.

The patients were randomly divided into control (n = 62) and experimental (n = 62) groups using a computerized randomization method. The baseline data between the 2 groups were compared, and no statistically significant differences were observed (*P* ˃ 0.05, Table 1).

**Table 1:** Comparison of baseline characteristics between 2 groups.

Baseline parameters	Control group (n = 62)	Study group (n = 62)	*t*/*χ*^2^	*P*-value
Gender (n, %)				
Male	30 (48.38%)	33 (53.22%)	0.29	.59
Female	32 (51.61%)	29 (46.77%)		
Age ($\bar{x}\pm s$, yr)	53.05 ± 15.69	51.60 ± 18.52	0.47	.64
Weight ($\bar{x}\pm s$, kg)	59.58 ± 11.83	63.05 ± 12.29	1.60	.11
Underlying condition (n, %)				
Hypertension	26 (41.93%)	31 (50.00%)		
Diabetes	14 (22.58%)	17 (27.41%)	2.51	.31
Others	22 (35.48%)	14 (22.58%)		

**Figure 1.**
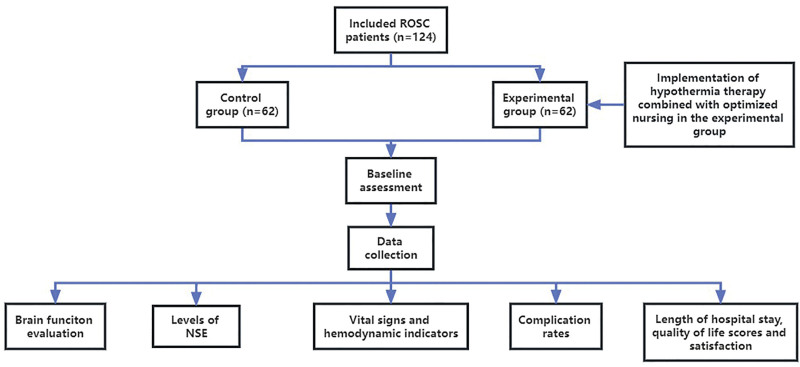
Flowchart of the study. NSE = neuron-specific enolase, ROSC = return of spontaneous circulation.

Procedure of study flowchart is presented in Figure 1.

### 2.2. Methods

The control group received routine care after CA/CPR. The routine care process was jointly conducted by the Emergency Department, Emergency Nurses, Imaging Department, and Emergency Laboratory Department of Fujian Provincial Hospital South Branch. The specific procedures were as follows:

Upon arrival, emergency personnel immediately conducted an on-site assessment and measurement of vital signs. Patients with CA received immediate CPR, including chest compressions, airway opening, endotracheal intubation, bag-valve-mask ventilation, and defibrillation for ventricular fibrillation. Vasoactive drugs like epinephrine and dopamine were administered.

#### 2.2.1. Post-resuscitation care

After CPR, patients received routine cooling therapy with ice caps to maintain the rectal temperature between 32 and 34 °C. After 24 hours, a slow rewarming was initiated. During this period, vital signs were closely monitored using cardiac monitoring. The criteria for successful CPR were the presence of carotid artery pulse after compressions were stopped, improvement in cyanosis of the face and lips, spontaneous breathing, constricted pupils, and mild reflex responses.

The experimental group received optimized emergency care combined with mild hypothermia management. Immediately after CPR, the patients were provided with cooling blankets and caps to achieve a target temperature of 32 to 34 °C within 3 hours. Rectal temperature was measured every 2 hours to ensure the maintenance of the desired range. Continuous infusion of propofol and remifentanil was administered for sedation and analgesia, with the medication dosage adjusted according to the patient’s body temperature. After 24 hours, slow rewarming increased the temperature by 0.2 to 0.5 °C/h to 36.5 to 37.4 °C. This temperature range was maintained for 72 hours after the ROSC. The optimized care also included additional treatments like reducing intracranial pressure, providing cerebral cell nutrition, correcting electrolyte imbalances, anti-infection measures, and mechanical ventilation support. Close observation of consciousness, pupil responses, bowel movements, and urine output was conducted during this process. Tracheal tube care was performed, and patients in the experimental group were repositioned every 2 hours to prevent pressure sores, frostbite, and aspiration pneumonia. Additionally, patients were verbally stimulated to assess their responsiveness. These modifications aim to improve post-resuscitation care by focusing on achieving and maintaining mild hypothermia and addressing the associated complications.

### 2.3. Observed indicators

#### 2.3.1. Consciousness

The Glasgow Coma Scale (GCS) was used for scoring consciousness levels, ranging from 3 to 15. A score of 3 to 8 indicated severe coma, 9 to 12 indicated moderate coma, and 13 to 14 indicated mild coma.

#### 2.3.2. Neurological function

The National Institutes of Health Stroke Scale (NIHSS)^[13]^ was utilized to assess neurological function, mainly recorded before and 72 hours after nursing care. It consisted of 11 components: visual field (0–3 points), gaze (0–2 points), sensation (0–2 points), speech (0–3 points), facial palsy (0–3 points), dysarthria (0–2 points), limb ataxia (0–2 points), upper-limb motor function (0–8 points), lower-limb motor function (0–8 points), consciousness level (0–7 points), and neglect (0–2 points). The total score ranged from 0 to 42, with higher scores indicating more severe neurological impairment.

#### 2.3.3. Brain injury indicators

The serum neuron-specific enolase (NSE) levels were measured in both groups of patients before and 72 hours after nursing care. The enzyme-linked immunosorbent assay method was used with reagent kits provided by the Wuhan Yunkelong Scientific and Technological Company.

#### 2.3.4. Vital signs

Changes in heart rate and blood oxygen saturation were observed in both groups of patients 72 hours after nursing care.

#### 2.3.5. Hemodynamic indicators

Invasive hemodynamic indicators, including mean arterial pressure (MAP), central venous pressure (CVP), and systolic blood pressure (SBP), were observed in both groups of patients 72 hours after nursing care.

#### 2.3.6. Complications

At 90 days post-discharge, patients may experience various complications, including electrolyte imbalances, pulmonary infections, kidney dysfunction, and stress-induced peptic ulcers. These complications have been observed to contribute to prolonged hospital stays and can lead to long-term implications such as limb mobility disorders, seizures, and speech disorders.

#### 2.3.7. Quality of life (QoL) score and satisfaction assessment

The Short Form Health Survey (SF36) was utilized to evaluate the QoL at 90 days post-discharge. This scale comprises 36 items that are further subdivided into 8 subscales, as described previously. Each subscale was assigned a score ranging from 0 to 100, where 0 represented the lowest QoL, and 100 signified the highest QoL. Furthermore, the satisfaction of the 2 nursing interventions was assessed for both groups.

#### 2.3.8. Statistical analysis

In this study, statistical analysis and graphical representation were performed using SPSS version 22.0 software (IBM SPSS, Armonk, NY, www-01.ibm.com/software/analytics/spss) and GraphPad Prism version 9.0.0 (GraphPad Software, San Diego, CA, http://www.graphpad.com). The Shapiro–Wilk normality test was used to assess the normal distribution of the data. For data that followed a normal distribution, mean ± standard deviation was used to represent the results, and the comparison between groups was conducted using the Student *t* test. For data that did not follow a normal distribution, median and interquartile range were used to represent the results, and nonparametric tests were employed for pairwise comparisons. Categorical data are presented as percentages (%), and the comparison between groups was analyzed using the chi-square test. In the statistical analysis, a *P*-value <.05 was considered statistically significant.

## 3. Results

### 3.1. Comparison of GCS and NIHSS scores between 2 groups

**Figure 2.**
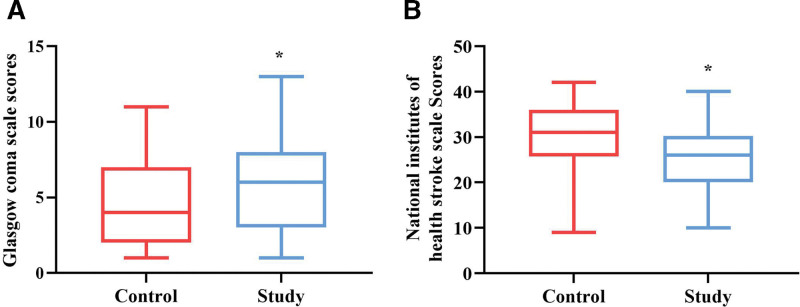
Evaluation of brain function in 2 groups. (A) GCS score (n = 62 per group). (B) NIHSS score. All data are presented as median and IQR (n = 62 per group). ^*^P < .05, compared to the control group. GCS = Glasgow Coma Scale, IQR = interquartile range, NIHSS = National Institutes of Health Stroke Scale.

At 72 hours post-ROSC, the level of consciousness and neurological function were assessed by assigning GCS and NIHSS scores to the control and experimental groups. The results showed a statistically significant difference in GCS scores between the control group (median: 4; Q1–Q3: 2–7) and the experimental group (median: 6; Q1–Q3: 3–8) after optimized nursing care was implemented (*P* ˂ .05, Fig. 2A). Similarly, the experimental group exhibited a significantly improved NIHSS score (median: 26; Q1–Q3: 20–30.25) than the control group (median: 31; Q1–Q3: 25.75–36) (*P* ˂ .05, Fig. 2B).

### 3.2. Comparison of serum NSE levels between the 2 groups

**Figure 3.**
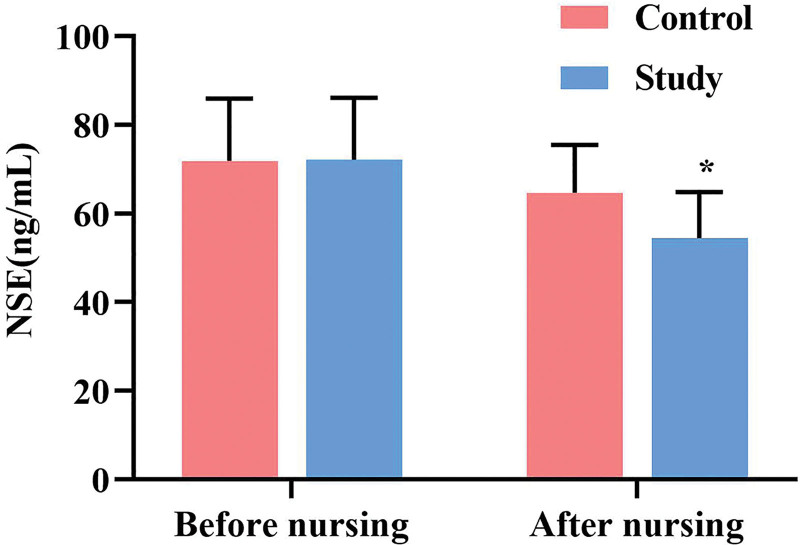
Serum NSE levels in 2 groups. Each group has a sample size of n = 30. All data are presented as $\bar{x}\pm s$. ^*^P < .05, compared to the control group. NSE = neuron-specific enolase.

The enzyme-linked immunosorbent assay results revealed no statistically significant difference in baseline serum NSE levels between the 2 patient groups prior to nursing care (*P* ˃ .05). However, after a 72-h period following ROSC, NSE levels decreased compared to baseline levels, and the experimental group demonstrated significantly lower NSE levels than the control group, indicating a statistically significant difference (*P* ˂ .05, Fig. 3).

### 3.3. Comparison of baseline vital signs between the 2 groups

**Figure 4.**
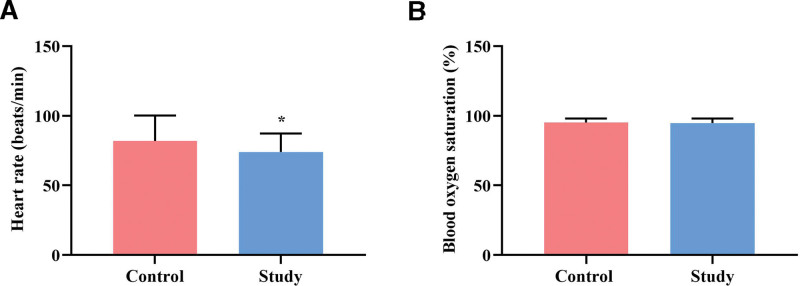
Comparison of baseline vital signs between 2 groups. (A) Heart rate (n = 62 per group). (B) Blood oxygen saturation (n = 62 per group). All data are presented as $\bar{x}\pm s$. ^*^P < .05, compared to the control group.

After 72 hours of nursing care, the experimental group had a significantly lower heart rate than the control group (*P* ˂ .05). However, no statistically significant difference was observed in blood oxygen saturation between the 2 groups (*P* ˃ .05, Fig. 4).

### 3.4. Comparison of hemodynamic monitoring results between the 2 groups

**Figure 5.**
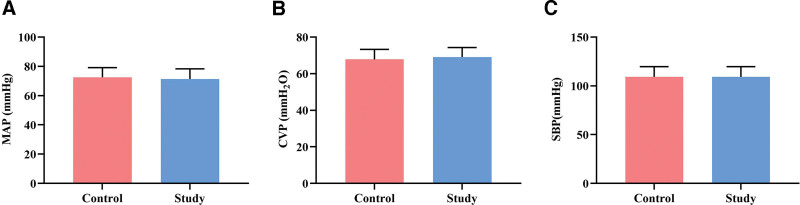
Comparison of hemodynamic monitoring results between 2 groups. Each group has a sample size of n = 62. All data are presented as $\bar{x}\pm s$. CVP = central venous pressure, MAP = mean arterial pressure, SBP = systolic blood pressure.

After 72 hours of optimized care, the invasive hemodynamic monitoring results showed no significant differences in MAP, CVP, and SBP between the 2 groups (*P* ˃ .05, Fig. 5).

### 3.5. Comparison of complication rates between the 2 groups

Following the nursing intervention, the control and experimental groups had complication rates of 95.16% (59/62) and 79.03% (49/62), respectively. The difference was statistically significant (*P* ˂ .05, Table 2). The results of the follow-up on long-term complications 90 days after discharge showed that the experimental group had significantly fewer long-term complications than the control group after optimized care (*P* ˂ .05, Table 3).

**Table 2:** Comparison of complication rates between 2 groups (n, %)

Groups	Electrolyte metabolic imbalance	Lung infection	Kidney dysfunction	Stress-induced peptic ulcer	Others	Overall incidence rates
Control group (n = 62)	25 (40.32%)	10 (16.13%)	9 (14.52%)	6 (9.68%)	9 (14.52%)	59 (95.16%)
Study group (n = 62)	22 (35.48%)	8 (12.90%)	8 (12.90%)	4 (6.45%)	7 (11.29%)	49 (79.03%)
*χ* ^2^						7.18
*P*-value						.01

**Table 3:** Comparison of long-term complications (n, %).

Groups	Motor impairment	Seizures	Speech disorder	Overall incidence rates
Control group (n = 62)	19 (30.65%)	8 (12.90%)	7 (11.29%)	34 (54.84%)
Study group (n = 62)	13 (20.97%)	4 (6.45%)	3 (4.84%)	20 (32.26%)
*χ* ^2^				6.43
*P*-value				.02

### 3.6. Comparison of length of hospital stay, QoL scores, and satisfaction between the 2 groups

**Figure 6.**
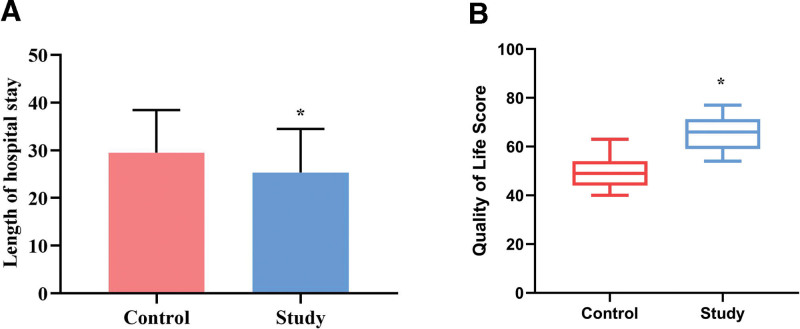
Comparison of length of hospital stay and quality of life scores between the 2 groups. Each group has a sample size of n = 62. The left graph represents the data presented as $\bar{x}\pm s$, while the right graph represents the data presented as median and IQR. ^*^P < .05, compared to the control group. IQR = interquartile range.

**Figure 7.**
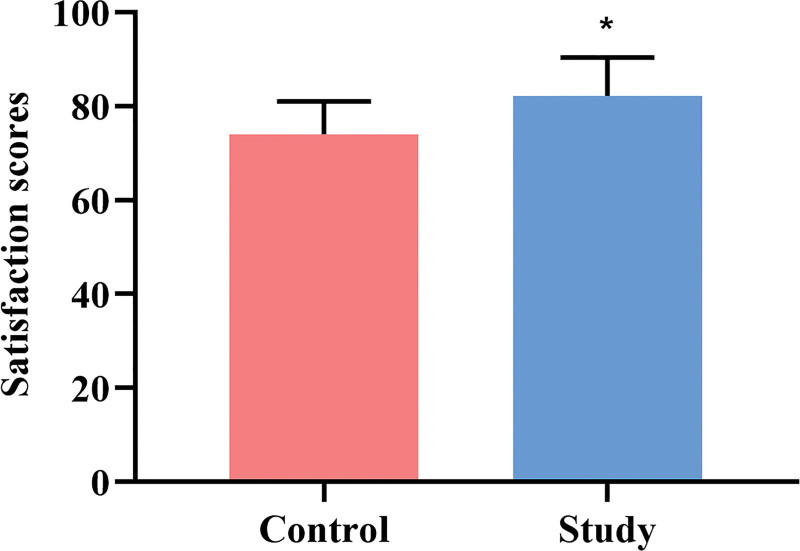
Comparison of satisfaction scores between 2 groups. Each group has a sample size of n = 62. All data are presented as $\bar{x}\pm s$. ^*^P < .05, compared to the control group.

The length of hospital stay was also compared between the 2 groups, with the control group having an average stay of 29.53 ± 8.92 days and the experimental group having 25.37 ± 9.10 days. There was a statistically significant difference between the 2 groups (*P* < .05, Fig. 6A). At 90 days post-discharge, the QoL scores were evaluated and compared between the control group (median: 49; Q1–Q3: 44–54) and the study group (median: 66; Q1–Q3: 59–71), showing a statistically significant difference between the 2 groups (*P* < .05, Fig. 6B). Additionally, the satisfaction scores after discharge were 74.02 ± 7.0 in the control group and 82.19 ± 8.2 in the study group, with a statistically significant difference noted between the 2 groups (*P* < .05, Fig. 7).

## 4. Discussion

In the post-resuscitation period after CA, brain injury is a major risk factor for disability and death in patients with CA.^[14]^ Studies have revealed that a higher body temperature during the recovery phase can cause damage to the patient’s brain function and increase adverse neurological outcomes.^[15,16]^ Therefore, to prevent further damage to brain function and to ensure the effectiveness of treatment after ROSC, active cooling care should be provided in clinical practice.

In this study, the results showed that the experimental group had significantly better GCS and NIHSS scores than the control group. The serum NSE results also confirmed that optimized emergency care combined with MTH can reduce brain tissue damage and improve neurological outcomes. This might be due to the more stringent temperature control provided by the optimized care, which enhances the neuroprotective effects of hypothermia after resuscitation. The combination of CPR and MTH in post-resuscitation care may involve the following mechanisms: (1) decreased adenosine triphosphate consumption rate: hypothermia slows down cellular energy consumption, offering protection to brain cells; (2) inhibition of excitatory neurotransmitter release: hypothermia decreases the release of excitatory neurotransmitters (like glutamate), thereby reducing damage to brain cells; (3) alteration of intracellular signaling: hypothermia can modify the activity of intracellular signaling molecules, exerting a protective effect; (4) attenuation of inflammatory response and blood-brain barrier disruption: hypothermia can mitigate the associated inflammatory response and protect the integrity of the blood-brain barrier; (5) modulation of gene expression and protein synthesis: hypothermia may regulate cell function and protective mechanisms by modifying gene expression and protein synthesis.^[17]^ Additionally, MTH can sedate the central nervous system by increasing ethanol (a neuroinhibitory agent) levels, leading to a sleep-like state in patients.^[18]^ This reduces oxygen consumption and metabolism and alleviates brain and pulmonary edema, thereby improving microcirculation and cardiopulmonary function. Therefore, optimized care and MTH effectively maintain a low-temperature state in patients, reduce the metabolic rate of the nervous system, minimize neuronal damage caused by anaerobic metabolism during CA, and provide positive protection for neurological recovery and prognosis.

Hypothermic care at subnormal temperatures helps protect brain tissue by reducing cerebral oxygen metabolism, cellular oxygen consumption, and oxygen content. It also inhibits oxygen free radicals and alleviates brain cell necrosis and apoptosis.^[19,20]^ Previous studies have shown that for every 1 °C decrease in body temperature, the metabolic rate decreases by 6% to 7%.^[21]^ In the experimental group, after 72 hours of care, the heart rate was lower compared to the control group. At the same time, there were no statistically significant differences in blood oxygen saturation, MAP, CVP, and SBP between the 2 groups. Since most post-ROSC patients required mechanical ventilation, the respiratory rate was not statistically analyzed. These findings indicate that adopting an intensified MTH care approach with strict temperature control within the range of 32 to 34 °C can mildly decrease the patient’s heart rate without adversely affecting their hemodynamics and cardiopulmonary function.

Moreover, serum NSE levels are elevated following brain injury and have been recommended for predicting the prognosis of brain function in patients with CA.^[22]^ The NSE results in both groups suggest that hypothermic care can further enhance the neuroprotective effects of MTH. This partially indicates that optimizing nursing interventions combined with MTH treatment in patients with brain injury after CPR may improve temperature regulation during diagnosis and treatment.

Optimizing nursing care is crucial for reducing complications and improving patient outcomes. By implementing central temperature MTH and closely monitoring vital signs, healthcare professionals can proactively identify and manage early symptoms, thereby minimizing complications.^[23]^ Through the proper use of instruments and timely adjustments of cooling blankets and ice caps, the patient’s body temperature can be controlled within the optimal range of 32 to 34 °C. The precise control of body temperature through MTH is better than conventional nursing methods. This approach provides a tailored and targeted therapy that helps maintain physiological homeostasis and supports the body’s natural healing processes. By avoiding temperature fluctuations and ensuring a stable environment, MTH reduces the risk of cardiac arrhythmias and electrolyte imbalances that are potentially harmful to patient recovery.

Besides temperature control, nursing staff are vital in providing comprehensive care to patients. They emphasize preventive measures, like appropriate dietary interventions, to prevent pressure ulcers and promote healing.^[24]^ These dietary interventions support the body’s nutritional needs, enhance tissue regeneration, and strengthen the immune system, thus reducing the likelihood of complications.^[23]^ Furthermore, the significant impact of patient interaction cannot be overlooked. Nursing staff engage in physical contact and verbal stimuli, which provide comfort, help stimulate the patient’s senses, and promote cognitive functioning. These interactions contribute to promoting social and emotional well-being, preventing cognitive decline, and ultimately improving patient satisfaction during hospital stays. The optimization of nursing care encompasses various aspects, including temperature control, comprehensive care, and patient interaction. These efforts can minimize complications and improve patient outcomes. By implementing evidence-based nursing practices, healthcare professionals can provide a holistic approach to care that ultimately contributes to improved patient recovery, shorter hospital stays, and increased patient satisfaction.

However, this study has limitations due to its retrospective design and the inclusion of subjects from a single hospital, which may restrict the generalizability of the results. Further prospective randomized controlled studies are needed to validate these findings and determine the optimal treatment strategies. The relationship between certain factors and the outcomes may not be fully explained due to the incomplete nature of patient medical records. Moreover, the study did not provide long-term follow-up results for patients. Long-term follow-up is crucial for a better evaluation of treatment effectiveness and long-term patient prognosis. Additionally, despite anonymous processing, it remains vital to ensure the protection of patient privacy and data security. However, this study aims to explore the effects of optimized emergency care combined with hypothermic therapy on the post-ROSC outcomes of patients with CA, with the hope of providing valuable information and guidance for clinical nursing practice.

## 5. Conclusions

In summary, the combination of optimized emergency care and hypothermic therapy can effectively improve the neurological prognosis of patients with CA, reduce physical complications, and enhance patient satisfaction.

## Acknowledgments

We are very grateful for the convenience provided by the data platform of Fujian Provincial Hospital for collecting data.

## Author contributions

**Conceptualization:** Zheng Gong, Xiahong Tang.

**Data curation:** Lirong Qiu, Xiahong Tang, Yan You, Yaxu Zhang.

**Investigation:** Zheng Gong.

**Methodology:** Zheng Gong, Xiahong Tang.

**Project administration:** Zheng Gong.

**Software:** Yan You.

**Supervision:** Xiahong Tang.

**Writing – original draft:** Yan You.

**Writing – review & editing:** Yan You.
